# A 20-Gene Set Predictive of Progression to Severe Dengue

**DOI:** 10.1016/j.celrep.2019.01.033

**Published:** 2019-01-29

**Authors:** Makeda Robinson, Timothy E. Sweeney, Rina Barouch-Bentov, Malaya Kumar Sahoo, Larry Kalesinskas, Francesco Vallania, Ana Maria Sanz, Eliana Ortiz-Lasso, Ludwig Luis Albornoz, Fernando Rosso, Jose G. Montoya, Benjamin A. Pinsky, Purvesh Khatri, Shirit Einav

**Affiliations:** 1 Department of Medicine, Division of Infectious Diseases and Geographic Medicine, Stanford University, Stanford, CA, USA; 2 Department of Microbiology and Immunology, Stanford University School of Medicine, Stanford, CA, USA; 3 Institute for Immunity, Transplantation, and Infection, Department of Medicine, Stanford University, Stanford, CA, USA; 4 Department of Medicine, Division of Biomedical Informatics Research, Stanford University, Stanford, CA, USA; 5 Department of Pathology, Stanford University School of Medicine, Stanford, CA, USA; 6 Clinical Research Center, Fundación Valle del Lili, Cali, Colombia; 7 Pathology and Laboratory Department, Fundación Valle del Lili, Cali, Colombia; 8 Department of Internal Medicine, Division of Infectious Diseases, Fundación Valle del Lili, Cali, Colombia

**Keywords:** transcriptomics, multi-coherent analysis, prognostics, biomarkers, severe dengue

## Abstract

There is a need to identify biomarkers predictive of severe dengue. Single-cohort transcriptomics has not yielded generalizable results or parsimonious, predictive gene sets. We analyzed blood samples of dengue patients from seven gene expression datasets (446 samples, five countries) using an integrated multi-cohort analysis framework and identified a 20-gene set that predicts progression to severe dengue. We validated the predictive power of this 20-gene set in three retrospective dengue datasets (84 samples, three countries) and a prospective Colombia cohort (34 patients), with an area under the receiver operating characteristic curve of 0.89, 100% sensitivity, and 76% specificity. The 20-gene dengue severity scores declined during the disease course, suggesting an infection-triggered host response. This 20-gene set is strongly associated with the progression to severe dengue and represents a predictive signature, generalizable across ages, host genetic factors, and virus strains, with potential implications for the development of a host response-based dengue prognostic assay.

## Highlights

• A multi-cohort analysis framework revealed a 20-gene set to predict severe dengue
• The 20 genes effectively predict severe dengue in existing and prospective cohorts
• The 20-gene set is generalizable across ages, host genetic factors, and virus strains
• The 20-gene set has potential implications for the development of a prognostic assay

## Graphical Abstract

**Figure:**
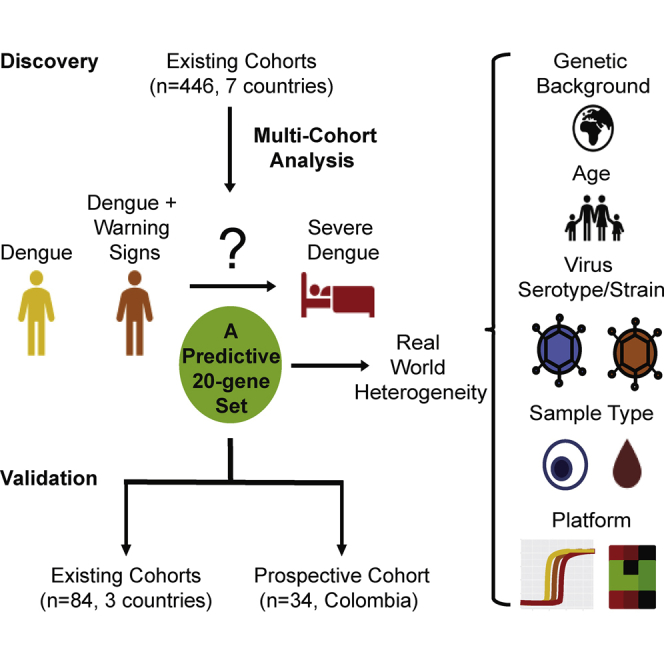

## Teaser

Biomarkers predictive of severe dengue are needed. Robinson et al. revealed a 20-gene set that predicts progression to severe dengue in existing and prospective cohorts via a multi-cohort analysis framework. This 20-gene set is generalizable across ages and host genetic factors and has potential implications for prognostic assay development.

## Introduction

About 400 million individuals annually are infected with any of the four dengue virus (DENV) serotypes (Bhatt et al., 2013). Although the majority of symptomatic individuals present with acute dengue fever, a fraction (∼5%–20%) of these patients progress to severe dengue manifested by bleeding, plasma leakage, shock, organ failure, and sometimes death (Khursheed et al., 2013, Thein et al., 2011, WHO, 2009). The greatest risk factor for severe dengue is secondary infection with a heterologous DENV serotype causing antibody-dependent enhancement (ADE), with variable contribution of aberrant activation of cross-reactive T cells (Katzelnick et al., 2017, Wang et al., 2017, Whitehorn and Simmons, 2011, Zivna et al., 2002). Early admission to an inpatient facility and administration of supportive care reduce mortality in patients with severe dengue (WHO, 2012). However, there are no usable prognostics to accurately predict which patients will progress to severe dengue. The 2009 World Health Organization (WHO) criteria classify dengue infection into uncomplicated dengue (D), dengue with warning signs (DWS), and severe dengue (SD), whereas the former (1997) criteria defined dengue fever (DF) and the two most common forms of severe dengue: dengue hemorrhagic fever (DHF) and/or dengue shock syndrome (DSS) (Kalayanarooj et al., 2017, WHO, 1997, WHO, 2009). Although improved, the currently utilized warning signs to identify patients at risk of progressing to severe dengue are clinical parameters that often develop late during the course of disease (Alexander et al., 2011, Srikiatkhachorn et al., 2011) and still have limited sensitivity and specificity, resulting in ineffective patient triage and resource allocation and continued morbidity and mortality (Kalayanarooj et al., 2017, Leo et al., 2013, Nujum et al., 2014, Thein et al., 2011).

Several attempts have been made to identify biomarkers associated with the development of severe dengue via microarray-based whole-genome analysis of host gene expression profiles in human peripheral blood (Devignot et al., 2010, Hoang et al., 2010, Kwissa et al., 2014, Loke et al., 2010, Long et al., 2009, Nascimento et al., 2009, Popper et al., 2012, Simmons et al., 2007, Sun et al., 2013, van de Weg et al., 2015). These studies identified differences in the timing and magnitude of gene transcript abundance, which were associated with disease severity (Loke et al., 2010, Popper et al., 2012). However, none of the existing gene sets have yet been shown to be generalizable. Nevertheless, these studies were deposited in publicly accessible databases, presenting novel opportunities for their re-analysis and re-use.

We previously developed an integrated multi-cohort analysis framework that integrates biologically heterogeneous datasets to identify robust host gene signatures that are generalizable and prospectively validated. We have used this framework to identify discrete diagnostic or prognostic gene sets in sepsis, viral infections, active tuberculosis, organ transplant, vaccination, and systemic sclerosis (Andres-Terre et al., 2015, Khatri et al., 2013, Lofgren et al., 2016, Sweeney et al., 2015, Sweeney et al., 2016a, Sweeney et al., 2016b). We hypothesized that integration of gene expression data from heterogeneous patient populations with dengue infection across a wide variety of ages, countries, and inclusion criteria would yield a set of conserved genes that is predictive of severe dengue and generalizable across cohorts.

## Results

### *In Silico* Discovery and Validation of a 20-Gene Set Predictive of Severe Dengue in Existing Cohorts

**Figure 1:**
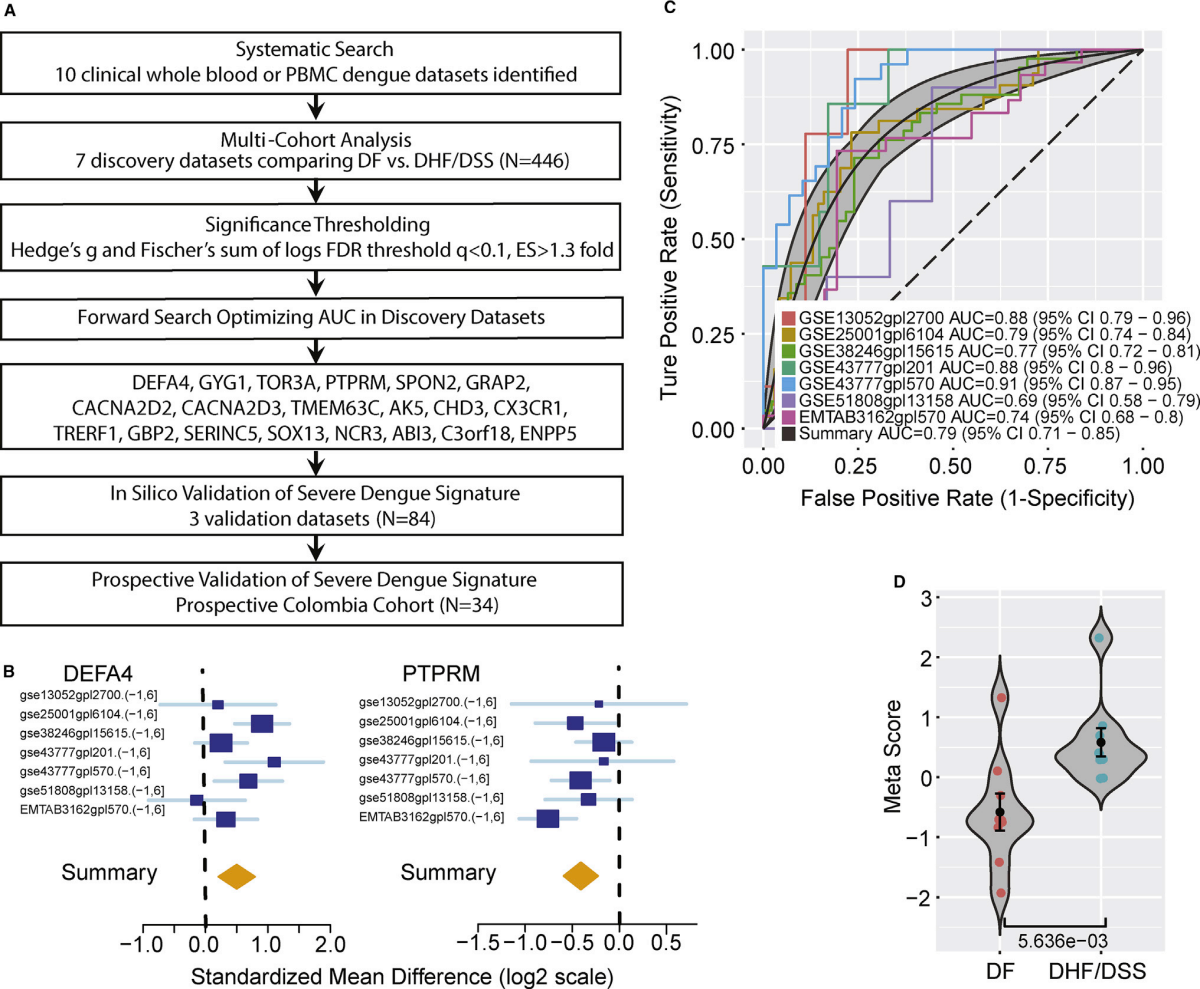
Discovery of the 20-Gene Set Predictive of Severe Dengue (A) Schematic of the multi-cohort analysis workflow for the discovery and validation of the 20-gene set. (B) Representative forest plots of over-expressed (DEFA4; left) and under-expressed (PTPRM; right) genes derived in the forward searches. The x axis represents standardized mean difference between DHF/DSS and dengue fever (DF). The size of the blue rectangles is inversely proportional to the SEM in the study. Whiskers represent the 95% CI. The orange diamonds represent overall, combined mean difference for a given gene. The width of the diamonds represents the 95% CI of overall combined mean difference. (C) ROC curves comparing patients with dengue fever with patients with DHF and/or DSS in the 7 discovery datasets. (D) A representative violin plot showing the performance of the 20-gene set for separating DHF and/or DSS from dengue fever in one of the discovery cohorts (GSE13052-GPL2700). Wilcoxon p value is shown. Error bars represent middle quartiles. ROC, receiver operating characteristic; FDR, false discovery rate; AUC, area under the curve.

We performed a systematic search for whole-genome expression datasets that examined whole blood or peripheral blood mononuclear cells (PBMCs) from patients with acute dengue infection. We identified 10 datasets and divided them into 7 “discovery” (Hoang et al., 2010, Kwissa et al., 2014, Loke et al., 2010, Long et al., 2009, Popper et al., 2012, Sun et al., 2013, van de Weg et al., 2015) and 3 “validation” (Devignot et al., 2010, Nascimento et al., 2009, Simmons et al., 2007) datasets, using samples obtained at admission prior to the development of severe dengue (Table S1). Using our multi-cohort analysis framework, we identified 59 significantly differentially expressed genes (false discovery rate [FDR] < 10%, effect size > 1.3-fold) between patients who progress to DHF and/or DSS (DHF/DSS) versus patients with an uncomplicated course (dengue fever) in the seven discovery datasets (N = 446) (Figure 1A). We applied an iterative greedy forward search (Sweeney et al., 2015) to the 59 genes and identified a set of 20 differentially expressed genes (3 over-expressed, 17 under-expressed) in DHF/DSS that was optimized for prognostic power (Figures 1B and S1; Table S2). We calculated a dengue score for each sample by subtracting the geometric mean expression of the 17 under-expressed genes from the geometric mean expression of the 3 over-expressed genes. The 20-gene dengue severity scores distinguished DHF/DSS from dengue fever upon presentation and prior to the onset of severe complications with a summary area under the curve (AUC) = 0.79 [95% confidence interval (CI) 0.71−0.85] in the discovery datasets (Figures 1C, 1D, and S2A).

**Figure 2:**
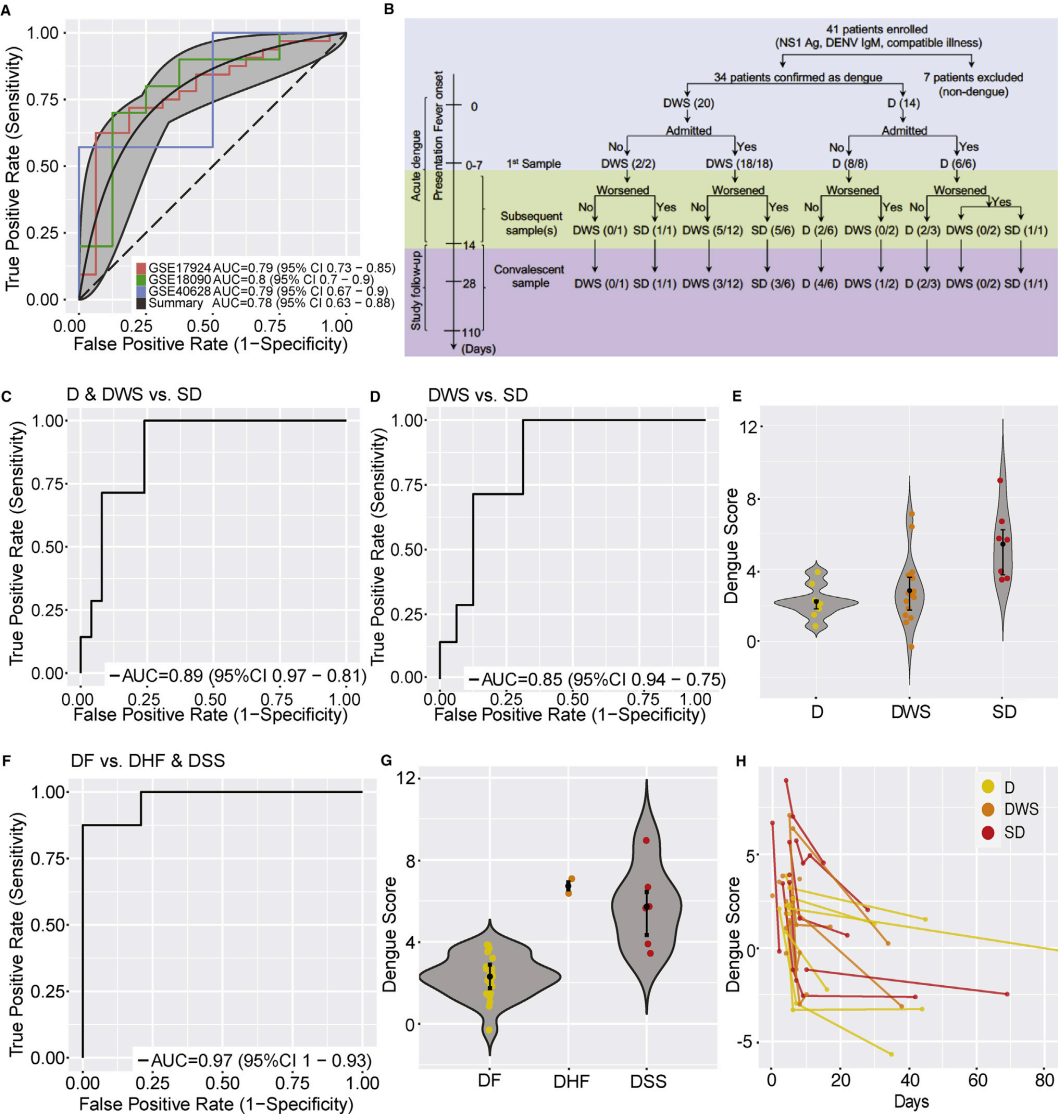
*In Silico* and Prospective Validation of the 20-Gene Set (A) ROC curves comparing patients with DHF and/or DSS with dengue fever patients in the 3 existing validation datasets. (B) Schematic of patient enrollment and sample collection in the prospective Colombia cohort. In parentheses are the number of samples available for each disease category/the number of patients in each disease category. (C) ROC curve comparing patients with severe dengue (SD) with patients with dengue with warning signs (DWS) or without (D) warning signs in the Colombia cohort. (D) ROC curve comparing patients with severe dengue with patients with dengue with warning signs in the Colombia cohort. (E) Violin plots showing the performance of the 20-gene set to separate severe dengue from dengue and dengue with warning signs in the Colombia cohort. (F) ROC curve comparing patients with DHF and/or DSS with dengue fever (1997 WHO criteria). (G) Violin plots showing the performance of the 20-gene set to separate dengue fever from DHF and DSS in the Colombia cohort (1997 WHO criteria). (H) Dengue severity scores in longitudinal samples from individuals in the Colombia cohort over time. (E and G) Error bars represent middle quartiles.

Next, we validated this 20-gene signature in the validation datasets (N = 84) (Devignot et al., 2010, Nascimento et al., 2009, Simmons et al., 2007) (Table S1). Despite the significant clinical heterogeneity in these datasets, including in age, host genetic factors represented by country of origin, source of sample, and inclusion criteria, the 20-gene dengue scores accurately identified dengue patients who will develop DHF/DSS in all three datasets (summary AUC = 0.78 [95% CI 0.63−0.88]) (Figure 2A). Additionally, dengue scores were significantly higher in DHF/DSS patients than in those with dengue fever in two of the datasets (Wilcoxon p values: GSE17924, p = 8.7e-4; GSE18090, p = 3.4e-2), albeit in the third dataset, the dengue scores did not reach statistical significance because of small sample size for the control group (GSE40628, p = 1.0e-01) (Figure S2B).

### Validation of the 20-Gene Set in a Prospective New Cohort of Dengue Patients

To further validate this signature, we established a cohort of prospectively enrolled dengue patients in Colombia (“Colombia cohort”) (Tables S3, S4, and S5). Disease severity was classified on-site using 2009 WHO criteria upon presentation and discharge (WHO, 2009). Forty-one patients were enrolled based on clinical presentation compatible with dengue or dengue with warning signs (patients classified as having severe dengue upon presentation were excluded) and positive NS1 antigen and/or anti-DENV IgM antibody. Whole-blood and serum samples were obtained upon presentation and at various time points during the disease course and/or at convalescence (Figure 2B). qRT-PCR (Waggoner et al., 2013) and serological assays (Zhang et al., 2017) confirmed the diagnosis of DENV infection in 34 patients. Upon discharge, 9 of these patients were diagnosed with dengue, 17 with dengue with warning signs (including one with dengue-Zika co-infection), and 8 with severe dengue (including one with dengue-pseudomonas co-infection) (Table S5). IgG avidity testing (Zhang et al., 2017) distinguished primary (N = 12) from secondary (N = 21) dengue (Table S5). Seven patients were excluded from the study due to establishment of alternative diagnoses (Zika [5], leptospirosis [1], and acute viral illness with prior dengue exposure [1]). One patient with severe dengue had degraded RNA leading to PCR failure and was removed from further analyses.

The transcripts for individual genes were quantified by high-throughput microfluidic qRT-PCR assays (Cheow et al., 2015) in samples of confirmed dengue patients. The 20-gene dengue score distinguished severe dengue from dengue with or without warning signs (AUC = 0.89 [95% CI 0.81–0.97]) and even severe dengue from dengue with warning signs (AUC = 0.85 [95% CI 0.75–0.94]) (Figures 2C and 2D).

The 1997 WHO criteria (WHO, 1997) were used for dengue classification in the publicly available datasets, whereas the 2009 criteria (WHO, 2009) were used in the Colombia cohort. To account for this difference in diagnosis, we re-analyzed the Colombia cohort data after blindly classifying patients based on the 1997 WHO criteria. The 20-gene dengue score had an AUC = 0.97 [95% CI 0.93−1.0] to distinguish dengue fever from DHF/DSS (Figures 2F and 2G) when using the 1997 WHO criteria.

Because different technologies were used in the various datasets, it was not possible to define a single diagnostic threshold to be used across different cohorts. Nevertheless, because we aim to identify a gene set that would not miss any of the patients who would progress to severe dengue, we chose a sensitivity of 100% in each cohort and computed the corresponding specificity (76%–79% in the Colombia cohort) (Table S6).

Next, we assessed the utility of laboratory parameters included in the WHO dengue classification to predict progression to severe dengue in the Colombia cohort. High hematocrit and/or low platelet count upon presentation failed to identify patients at risk to develop severe dengue (hematocrit: AUC = 0.73 [95% CI 0.61−0.84]; platelets: AUC = 0.65 [95% CI 0.53−0.77]; hematocrit and platelets: AUC = 0.77 [95% CI 0.66−0.87]) (Figure S3A), in line with prior publications demonstrating no, low, or time-limited predictive power of these parameters (Lam et al., 2017, Leo et al., 2013). Combining these two laboratory values with the 20-gene set did not significantly increase the prognostic power of the latter (AUC = 0.91 [95% CI 0.83−0.98]) (Figure S3A).

The dengue severity scores negatively correlated with platelet count (R^2^ = 0.202, p = 0.008), but did not correlate with hematocrit peak, total leukocytes, and their subtypes nadir, viral load, or dengue exposure (Figure S3B). Notably, a single patient (number 10) with serological evidence for primary infection presented with severe dengue (Table S5). The DENV serotype did not appear to affect the dengue severity score, albeit the sample number for some serotypes was small (Figure S3B).

To determine whether this transcriptomic signature preceded the infection or was triggered by it, we monitored its dynamics in longitudinal samples collected from the Colombia cohort during the disease course and after clinical recovery. The dengue scores progressively declined over time (Figure 2H), suggesting that DENV infection itself triggered the higher scores measured in severe dengue patients.

### The 20-Gene Set Is Enriched in NK and NKT Cells

Next, we explored whether the 20-gene signature is enriched in certain immune cell types using a previously reported cell-type enrichment analysis that uses publicly available whole-genome expression profiles from 25 different types of immune cells (Sweeney et al., 2015, Sweeney et al., 2016a, Sweeney et al., 2016b). We found that the 20-gene set is significantly downregulated (p < 0.05) in natural killer (NK) and NK T (NKT) cells (Figure S4A). Applying immunoStates with support vector regression (Bongen et al., 2018, Roy Chowdhury et al., 2018, Vallania et al., 2018) in the discovery and validation cohorts revealed no statistically consistent and reproducible differences in the proportions of the 20 estimated immune cell types (data not shown), including NK cells (Figures S4B–4D), between dengue-infected patients who progressed to severe dengue and those experiencing an uncomplicated course. These results suggest that the enrichment of NK cells observed for the 20-gene signature is likely due to changes in their expression level, rather than in NK cell-population abundance.

## Discussion

We identified a 20-gene host response signature to dengue infection for predicting the progression to severe dengue upon the onset of dengue infection but prior to the development of complications. We validated this 20-gene set both in existing cohorts and in a prospective cohort from Colombia and demonstrated that it performs well under both the current and former WHO dengue classification methods. Our discovery and validation cohorts were from 8 countries on 3 continents, providing strong evidence that the 20-gene set is not modulated by the underlying genetic background of the patients or the virus strains. Arguably, the overall prospective validation sample size is limited. However, the prognostic power of the 20-gene signature is maintained across a broad and heterogeneous patient mix. To the best of our knowledge, this study analyzed DENV infection data from the largest number of countries, and identified a prognostic gene signature that is robust to biological and technical heterogeneity observed in a real-world patient population. In contrast, it remains to be determined whether other predictive markers of severe dengue, such as chymase level (Tissera et al., 2017) and IgG antibody subtype (Wang et al., 2017) or level (Katzelnick et al., 2017), work in such heterogeneous patient populations.

The 20-gene set performed equally in DENV-infected children and adults, suggesting that it is not affected by age-dependent variations in immune responses. It also performed well in several immunosuppressed patients and a pregnant patient with severe dengue, yet it unnecessarily predicted severe dengue in two early postpartum patients (Table S5). Larger cohort studies are required to determine its utility in these special populations.

The 2009 WHO criteria intentionally define severe dengue more broadly than the 1997 WHO criteria, which are specific to DHF/DSS, a condition resulting from vascular leak (Katzelnick et al., 2017). The 20-gene signature had higher accuracy in predicting progression to severe dengue when using the 1997 criteria (area under the receiver operating characteristic curve [AUROC] = 0.97 [95% CI 0.93−1.0]) compared to the 2009 WHO criteria (AUROC = AUC = 0.89 [95% CI 0.81–0.97]) in the same cohort. These results suggest that the 20-gene signature is likely associated with DHF/DSS and that larger studies are required to better define its role in predicting less common cases of severe dengue caused by mechanisms that are not linked to vascular leak (Kalayanarooj et al., 2017).

The roles of the 20 gene products in the pathogenesis of severe dengue will be studied in the future. Cell-type enrichment analysis and immunoStates analysis suggest that reduced expression of these genes in NK and NKT cells rather than reduced NK and NKT cell-population abundances may be involved in driving their under-expression in severe dengue, yet the gene set likely encodes information that incorporates multiple cell-type shifts. Gene suppression may enhance pathogenesis via impaired viral clearance and/or altered immune responses. Alternatively, it is possible that some of the under-expressed genes in the set represent antiviral factors whose collective suppression may promote DENV replication, thereby increasing dengue severity (Vaughn et al., 2000). Indeed, many of the 20-gene set products are implicated in various aspects of antiviral innate immune responses, including adhesion and/or opsonization (SPON2 and PTPRM, CX3CR1; Bianchi et al., 1999, Fong et al., 2002, He et al., 2004), oxidative killing (GBP2), NK cell function (CX3CR1, NCR3), and signaling (GRAP2, CACNA2D2, GBP2; Baril et al., 2013, Lin and Brass, 2013, Saito et al., 2004). CHD3, a chromatin-remodeling factor, may mediate its role in DENV infection by interacting with the nonstructural 3 and 5 DENV proteins (Le Breton et al., 2011).

Our 20-gene set predicts the progression to severe dengue early in the course of dengue infection with high sensitivity and specificity (100% and 76%–79% in the Colombia cohort, respectively) (Table S6) and is robust to clinical heterogeneity of DENV infection. These findings indicate that once validated in larger prospective cohorts, this signature could be potentially used as a molecular prognostic tool to help triage dengue patients and define their level of care, thereby reducing morbidity and mortality while allocating resources more effectively. This is particularly important in the setting of dengue outbreaks. By identifying patients at high risk to develop severe dengue, the population that will likely benefit the most from antiviral therapy, such an assay can also guide patient selection and possibly endpoint measurements in clinical trials aimed at evaluating emerging anti-DENV agents, as those we and others have been developing (Bekerman et al., 2017). Various platforms that enable multiplexing of a large number of genes based on either RT-PCR (Liu et al., 2011), nucleic acid amplification and a semiconductor biochip (Hassibi et al., 2018), or branched DNA (Knudsen et al., 2008) technologies are already available and others are being developed. These technologies could facilitate the development of a cost-effective sample-to-answer assay that can provide a result in a reasonable turnaround. Once such a point-of-care prognostic assay for measuring the 20 genes is developed, its variability will be determined experimentally to define a single sensitivity threshold to be used across patient populations. Additional machine learning will then be applied to further validate the predictive power of the 20-gene set. Nevertheless, we have previously shown that a geometric mean-based classifier performs equally well as or better than other modeling approaches (Khatri et al., 2013, Lofgren et al., 2016). In agreement with our prior experiences, our current results highlight the utility of the geometric mean-based gene score to identify robust gene signatures using a parameter-free classifier without requiring any adjustments to the model. This approach is particularly useful in this case, because different technologies were used to measure the genes in the various dengue cohorts.

In summary, our study reveals a set of 20 genes that are highly associated with the progression to severe dengue early in the disease. This gene expression prognostic approach should be considered for further validation in larger prospective cohorts that could be utilized for the development of the first prognostic assay for use in dengue endemic countries.

## STAR★Methods

### Key Resources Table

**Table:** 

REAGENT or RESOURCE	SOURCE	IDENTIFIER
**Biological Samples**		

Dengue Patient Whole Blood	Fundación Valle del Lili	Human Subjects in Medical Research (Protocol # 35460)
Dengue Patient Serum	Fundación Valle del Lili	Human Subjects in Medical Research (Protocol # 35460)

**Critical Commercial Assays**		

Paxgene RNA Kit	PreAnalytiX	CAT#762165
Biomark Microfluidic qPCR Array	Stanford University Human Immune Monitoring Center	Cheow et al. (2015). Multiplexed locus-specific analysis of DNA methylation in single cells. Nat Protocols 10, 619-631.
Dengue Duo Combo Test	SD. Bioline	CAT#11FK45
Plasmonic-gold IgG Avidity Test	Nirmidas Biotech	http://www.nirmidas.com/nirmidas-news/2017/3/1/pgold-zikadengue-iggiga-assay-available
Multiplex rRT-PCR for DENV Serotyping	Pinsky lab	Waggoner et al. (2013). Single-Reaction, Multiplex, Real-Time RT-PCR for the Detection, Quantitation, and Serotyping of Dengue Viruses. PLoS Negl Trop Dis 7, e2116.
ZCD Assay (Zika, Chikungunya, and Dengue RT-PCR)	Pinsky lab	Waggoner et al. (2016). Single-Reaction Multiplex Reverse Transcription PCR for Detection of Zika, Chikungunya, and Dengue Viruses. Emerg Infect Dis 22, 1295-1297.

**Deposited Data**		

Colombia dataset	This paper	GEO:GSE124046

**Software and Algorithms**		

MetaIntegrator	R package	https://cran.r-project.org/web/packages/MetaIntegrator/index.html
Cell Type Enrichment Analysis	R package	Sweeney et al. (2015). A comprehensive time-course–based multicohort analysis of sepsis and sterile inflammation reveals a robust diagnostic gene set. Science Translational Medicine 7, 287ra271.
Deconvolution Analysis (within MetaIntegrator)	R package	Vallania et al. (2018). Leveraging heterogeneity across multiple datasets increases cell-mixture deconvolution accuracy and reduces biological and technical biases. Nature Communications 9 (4735).
Prism 7	Graphpad	https://www.graphpad.com/scientific-software/prism/

### Contact for Reagent and Resource Sharing

Additional information and requests for resources and reagents should be directed to and will be fulfilled by the Lead Contact, Shirit Einav (seinav@stanford.edu).

### Experimental Model and Subject Details

#### Colombia cohort ethics statement

All work with human subjects was approved by the Stanford University Administrative Panel on Human Subjects in Medical Research (Protocol # 35460) and the Fundación Valle del Lili Ethics committee in biomedical research (Cali/Colombia). All Subjects, their parents or legal guardians provided written informed consent, and subjects between 6 to 17 years of age and older provided assent. Subjects were not involved in previous procedures and were all test naive. The health/immune status of the subjects are summarized in Table S5.

#### Study population and sample collection

Blood samples were collected from individuals presenting to the emergency room or clinics of the Fundación Valle del Lili in Cali (Colombia) between March 2016 and June 2017. Enrollment criteria consisted of: i) age greater than 2 years; ii) presentation with an acute febrile illness of less than 7 day duration associated with one or more of the following symptoms or signs: headache, rash, arthralgia, myalgia, retroorbital pain, abdominal pain, positive tourniquet test, petechiae, and bleeding; and iii) a positive dengue IgM antibody and/or NS1 antigen by the SD. BIOLINE Dengue Duo combo device (Standard Diagnostic Inc., Korea) test (Wang and Sekaran, 2010). Two patients with a clinical presentation highly consistent with dengue were enrolled in face of having negative DENV IgM and NS1 antigen.

Patients were classified by infectious diseases specialists as having dengue, dengue with warning signs or severe dengue according to 2009 WHO criteria (Alexander et al., 2011, WHO, 2009) upon both presentation and prior to their discharge (Figure 2B). Patients presenting with severe dengue were excluded from the study. 41 patients were enrolled and additional demographic information can be found in Table S3. Discharge diagnoses were also blindly classified by infectious diseases specialists according to the 1997 WHO criteria into dengue fever, DHF, and/or DSS criteria. Demographics and clinical information were collected at the time of presentation. The first day of fever (fever day 0) was defined by the patients or their relatives. Symptoms, signs, and laboratory studies (including complete blood count, chemistry, and liver function tests) were documented by healthcare professionals (Tables S4 and S5).

The first venous blood sample was collected upon enrollment on the first day of presentation (Figure 2B). Patients presenting with dengue with warning signs provided additional blood samples every 48 to 72 hours during their hospital admission. When possible, an additional sample was obtained from all patients following defervescence (1-17 weeks after the initial presentation) during a routine visit to the infectious diseases clinic (Figure 2B). 2.5 mL of whole blood were collected in Paxgene tubes (PreAnalytiX) and stored at −80°C. Serum samples were obtained for additional assays. Samples transport, reception, and processing were strictly controlled using personal data assistants (PDAs) with barcode scanners.

### Method Details

#### Establishment of dengue diagnosis

##### Detection of DENV NS1 antigen and IgG/IgM

The SD. BIOLINE Dengue Duo combo test (Standard Diagnostic Inc., Korea) (Wang and Sekaran, 2010), which is routinely used in the Department of Pathology

(Fundación Valle del Lili) was used to identify dengue patients for enrollment to the study.

##### qRT-PCR assays for detection of dengue and other microbial pathogens

To confirm the diagnosis of dengue and differentiate from infection with the co-circulating arboviruses, Zika virus and chikungunya virus, serum samples were screened with a qualitative, single-reaction, multiplex real-time reverse transcriptase PCR (rRT-PCR) that detects Zika, chikungunya, and dengue virus RNA (Waggoner et al., 2016). To identify the specific DENV serotype and determine the virus load, samples positive for DENV in the screening assay were serotyped and quantitated using a separate DENV multiplex rRT-PCR (Waggoner et al., 2013). A single sample was also subjected to rRT-PCR for leptospira.

#### Multiplexed serological assays on a plasmonic-gold platform

Multiplexed antigen microarrays including DENV-2 whole virus particles spotted in triplicate were fabricated on pGOLD slides (Nirmidas Biotech, California) and serologic testing performed, as described (Zhang et al., 2017). Briefly, for DENV IgG and IgM testing, each well was incubated with human sera (400 times dilution) for 40 min, followed by incubation of a mixture of anti-human IgG-IRDye680 conjugate and anti-human IgM-IRDye800 conjugate for 15 min (Vector-Laboratories, Burlingame, CA). Each well was washed between incubation procedures. The biochip was then scanned with a MidaScan-IR near-infrared scanner. IRDye680 and IRDye800 fluorescence images were generated, and the median fluorescence signal for each channel on each microarray spot was quantified by MidaScan software. For each sample, each antigen and each channel, the average of the three median fluorescence signals for three spots was calculated and normalized by positive and negative reference samples through a two-point calibration. Previously defined cutoffs based on mean levels +3 SD were used (Zhang et al., 2017).

DENV IgG avidity was performed as above in duplicate wells, except that following primary incubation, one well was incubated with 10 M urea for 10 min. Then, anti-human IgG-IRDye680 conjugate was applied to each well and incubated for 15 min. DENV IgG avidity was calculated by dividing the normalized DENV IgG result of the sample tested with urea treatment by the normalized DENV IgG result of the sample without urea treatment. High avidity (> 0.6) is indicative of a past infection, whereas low avidity (< 0.6) is consistent with a recent infection.

#### RNA extraction

RNA was extracted from PAXgene tubes using the PAXgene blood RNA extraction kit (QIAGEN) and analyzed for RNA quality using the Agilent bioanalyzer QC analysis.

#### Quantification of gene transcripts

##### High-throughput microfluidic qRT-PCR assays

The Biomark Microfluidic qPCR Array was used to quantify the individual transcripts of the signature at the Stanford Human Immune Monitoring Center, as previously described (Cheow et al., 2015). 50 ng of total RNA was reverse transcribed at 50°C for 15 minutes using the High Capacity Reverse Transcription kit (ABI). Preamplification was performed on a thermocycler following addition of the TaqMan PreAmp Master Mix Kit (Invitrogen) to the pooled Taqman assays and cDNA. RT enzyme was inactivated and the Taq polymerase reaction was initiated by bringing the sample to 95°C for 2 minutes. The cDNA was preamplified by denaturing for 10 cycles at 95°C for 15 s and annealing at 60°C for 4 minutes. The resulting cDNA product was diluted 1:2 with 1X TE buffer (Invitrogen). 2X Applied Biosystems Taqman Master Mix, Fluidigm Sample Loading Reagent, and preamplified cDNA were mixed and loaded into the 48.48 Dynamic Array (Fluidigm) sample inlets, followed by loading 10X Taqman gene expression assays into the assay inlets. Manufacturer’s instructions for chip priming, pipetting, mixing, and loading onto the BioMark system were followed. RT-PCR was carried out at the following conditions: 10 min at 95°C followed by 50 cycles of 15 s at 95°C and 1 min at 60°C. Data were analyzed using Fluidigm software. All reactions were performed in duplicate and Ct values were normalized to 18S RNA and beta-actin. TaqMan reagents are listed below.

### Quantification and Statistical Analysis

Systematic search and analysis. We searched two public gene expression microarray repositories (NIH GEO and ArrayExpress) for all human gene expression dengue datasets. We retained datasets that examined clinical cohorts of dengue infection in whole blood or PBMCs for further study, and excluded datasets that examined only dengue with no severe dengue, were done in patients on steroid treatment, were non-clinical (e.g., cell culture studies), or used on-chip two-sample arrays. The remaining 10 datasets contained 530 samples from 7 countries from both adult and pediatric patients (Table S1).

We compared gene expression in patients with dengue fever versus patients with DHF and/or DSS using our validated multi-cohort analysis framework, as previously described (Andres-Terre et al., 2015, Khatri et al., 2013, Lofgren et al., 2016, Sweeney et al., 2015, Sweeney et al., 2016a, Sweeney et al., 2016b). We used seven datasets as the discovery cohort, and three datasets were left out for independent validation. The discovery/validation split was made such that there was a similar proportion of whole blood to PBMCs, and similar spread across years.

GC Robust Multi-array Average (GCRMA) normalization was used for Affymetrix chips and a normal-exponential correction followed by quantile normalization for all other chip types. All arrays were log2 normalized prior to analysis. No inter-dataset normalization was performed since different technologies were used in the various datasets. We applied a DerSimonian-Laird random-effects model to combine gene expression effect sizes via Hedges’g. We chose DerSimonian-Laird because of our previously published analysis of various random effects inverse variance models across a range of diseases (Sweeney et al., 2017) that showed DerSimonian-Laird provided good compromise to identify differentially expressed genes while reducing false positives. We set significance thresholds for differential expression at FDR less than 10% and an effect size greater than 1.3 fold (in non-log space). These thresholds for gene selection come from our prior analysis of different meta-analysis models (Sweeney et al., 2017).

#### Derivation of dengue score

To identify a parsimonious gene set maximized for diagnostic power, we began by running a forward search, using the MetaIntegrator R package, as previously described (Haynes et al., 2017, Sweeney et al., 2016a). Briefly, the algorithm starts with the single gene with the best discriminatory power, and then at each subsequent step adds the gene with the best possible increase in weighted AUC (area under the curve; the sum of the AUC for each dataset times the number of samples in that dataset) to the set of genes, until no further additions can increase the weighted AUC more than some threshold amount (here 0.005 × the total number of samples). At each iteration of the greedy forward search, when adding a new gene, we defined a dengue score as follows: for each sample, the mean expression of the downregulated genes is subtracted from the mean expression of the upregulated genes to yield a dengue score.

Since there was a substantial amount of clinical heterogeneity present, we wanted to maximize the diagnostic performance rather than aiming for extreme parsimony. Thus, we ran the forward search exhaustively, such that once a gene set had been identified, those genes were removed from the remaining pool and the forward search was run again. We set an arbitrary minimum threshold for performance of a mean AUC of 0.75 in the discovery data, which yielded three gene sets with a total of 20 genes. The entire list of 20 genes was then pooled to make a single dengue score. This dengue score was tested for diagnostic power using receiver operating characteristic (ROC) curves.

#### Validation of dengue score

We validated the 20-gene set in three independent clinical dengue gene expression datasets, comparing its ability to differentiate dengue fever from DHF/DSS.

Between-groups dengue score comparisons were done with the Wilcoxon rank sum test. Significance levels were set at two-tailed p < 0.05, unless specified otherwise.

All computation and calculations were done in the R language for statistical computing (version 3.0.2).

As previously reported (Sweeney et al., 2015, Sweeney et al., 2016a), we searched GEO for human immune cell type–specific gene expression profiles and found 277 samples from 18 datasets matching our criteria. We aggregated these into broad immune cell type signatures using mean gene expression scores. We then calculated standardized enrichment scores using the same method as the infection z score (difference of geometric means between positive and negative genes).

##### Deconvolution analysis

We performed cell-mixture deconvolution analysis as previously described (Vallania et al., 2018). Briefly, we first converted each microarray dataset into a gene-expression matrix, which was then deconvolved using immunoStates with a linear regression model (Vallania et al., 2018). A Hedge’s g effect size was then computed to estimate changes in cell subset proportions. Effect sizes from all individual datasets were integrated into a summary effect size and significance was computed as previously described (Haynes et al., 2017, Khatri et al., 2013). Analysis and plots were generated using the R programming language.

### Data and Software Availability

The Colombia cohort transcriptomic dataset was posted publicly. Please, see Gene Expression Omnibus (GEO) accession number:GSE124046 (scheduled to be released on Feb 04, 2019).

## Supplemental Information

Document S1. Figures S1–S4 and Tables S1–S4 and S6

Table S5. Colombia Cohort: Demographic and Clinical Characteristics of the Study Population, Related to Figure 2

Document S2. Article plus Supplemental Information
