# Supplementary material for: A 20-Gene Set Predictive of Progression to Severe Dengue
Source: Cell Rep. 2019 Jan 29;26(5):1104–1111.e4. doi: 10.1016/j.celrep.2019.01.033 (PMC6352713; doi:10.1016/j.celrep.2019.01.033)
Supplement: Document S1. Figures S1–S4 and Tables S1–S4 and S6 [file mmc1.pdf]

Cell Reports, Volume *26*

Supplemental Information

A 20-Gene Set Predictive

of Progression to Severe Dengue

Makeda Robinson, Timothy E. Sweeney, Rina Barouch-Bentov, Malaya Kumar Sahoo, Larry Kalesinskas, Francesco Vallania, Ana Maria Sanz, Eliana Ortiz-Lasso, Ludwig Luis Albornoz, Fernando Rosso, Jose G. Montoya, Benjamin A. Pinsky, Purvesh Khatri, and Shirit Einav

Supplemental Information

Supplemental figures

Figure S1. Forest plots of the over-expressed and under-expressed genes derived in the forward searches, Related to Figure 1.

Figure S2. Violin plots showing the performance of the 20-gene set to separate DHF/DSS from DF in the 7 datasets of the discovery cohort (A) and 3 datasets of the validation cohort (B), Related to Figure 1.

Figure S3. Routine laboratory parameters are ineffective in predicting development of severe dengue and at most part do not correlate with the dengue severity score, Related to Figure 2.

Figure S4. Enrichment profiles of the 20 differentially expressed genes in publicly available sorted-cell gene expression profiles and meta-analysis of cell-mixture deconvolution, Related to Figure 1.

Supplemental tables

Table S1. Publicly available datasets used for the discovery and validation of the 20-gene set, Related to Figures 1 and 2.

Table S2. Over-expressed and under-expressed genes identified in the discovery cohort via the multi-cohort analysis, Related to Figure 1.

Table S3. Colombia cohort. Demographic, clinical, and laboratory characteristics of study population, Related to Figure 2.

Table S4. Severe dengue patients in the Colombia cohort, Related to Figure 2.

Table S5. Demographic and clinical characteristics of the Colombia cohort study population. Excel file, Related to Figure 2.

Table S6. Sensitivity and specificity of the 20 gene-set in the pre-existing cohorts and new Colombia cohort, Related to Figures 1 and 2.

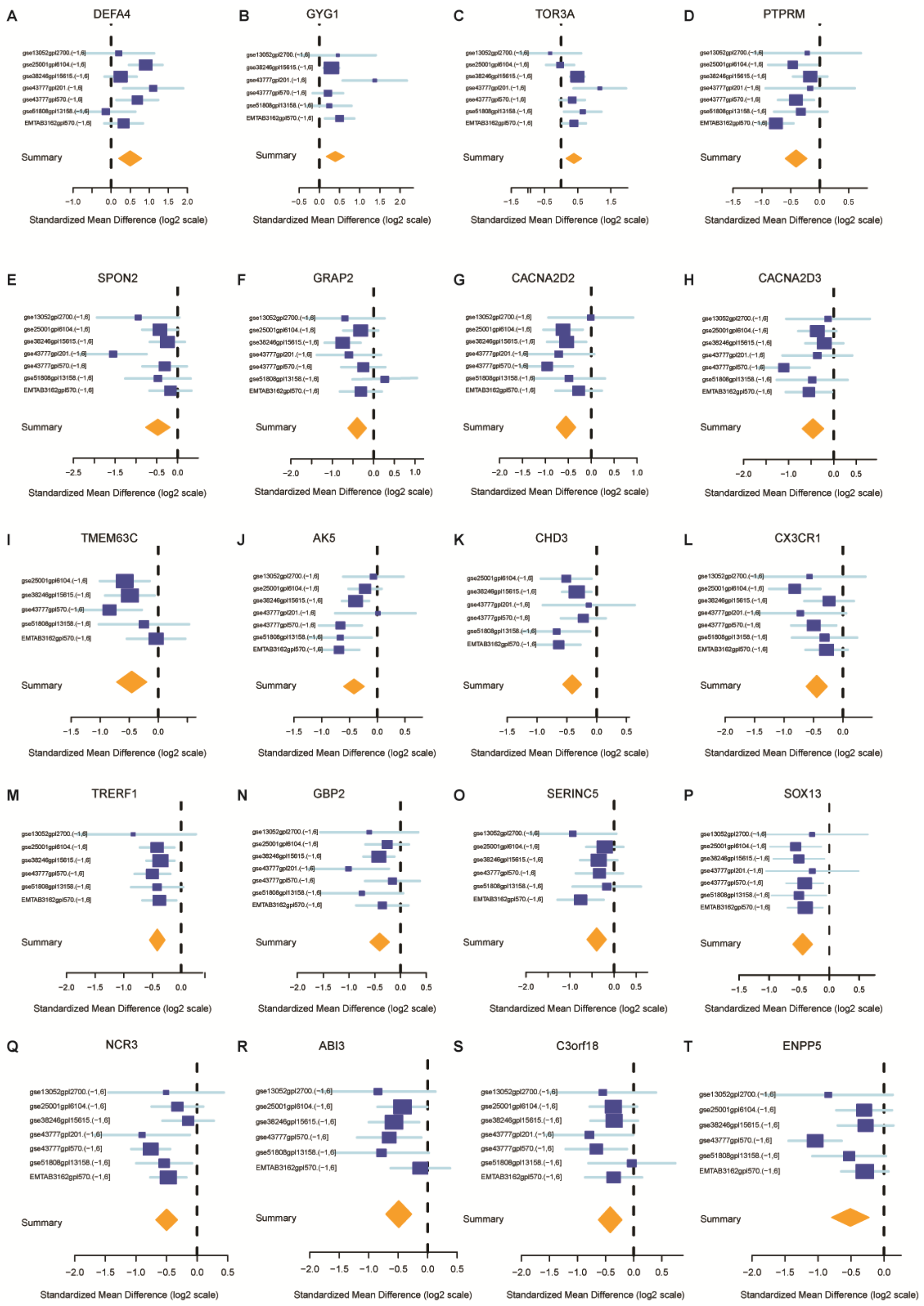

Figure S1. Forest plots of the over-expressed and under-expressed genes derived in the forward searches, Related to Figure 1. The x axis represents standardized mean difference between DHF/DSS and DF. The size of the blue rectangles is inversely proportional to the standard error of mean in the study. Whiskers represent the 95% CI. The orange diamonds represent overall, combined mean difference for a given gene. Width of the diamonds represents the 95% CI of overall combined mean difference.

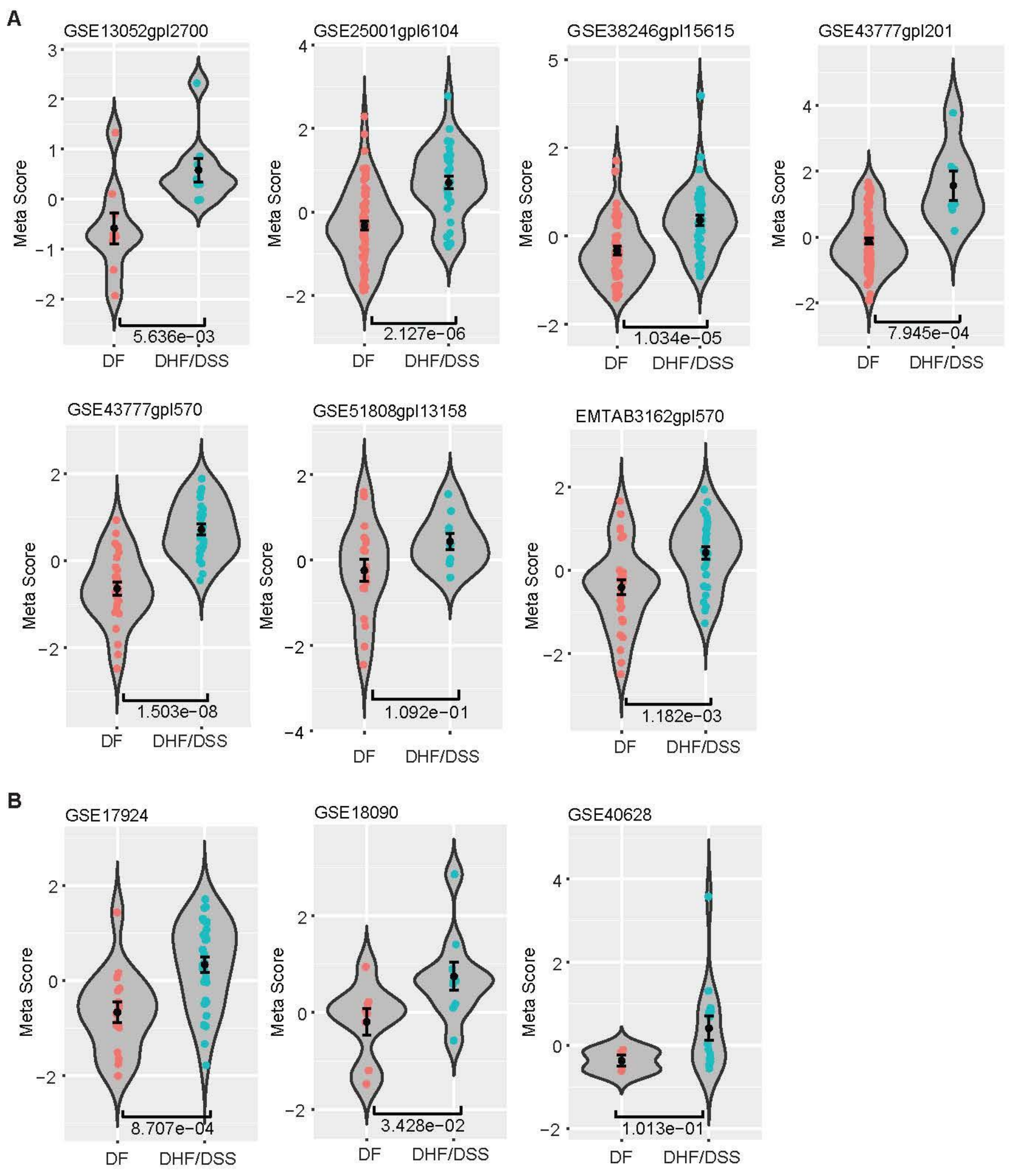

Figure S2. Violin plots showing the performance of the 20-gene set to separate DHF/DSS from DF in the 7 datasets of the discovery cohort (A) and 3 datasets of the validation cohort (B), Related to Figure 1. Wilcoxon P values shown. The error bars represent middle quartiles.

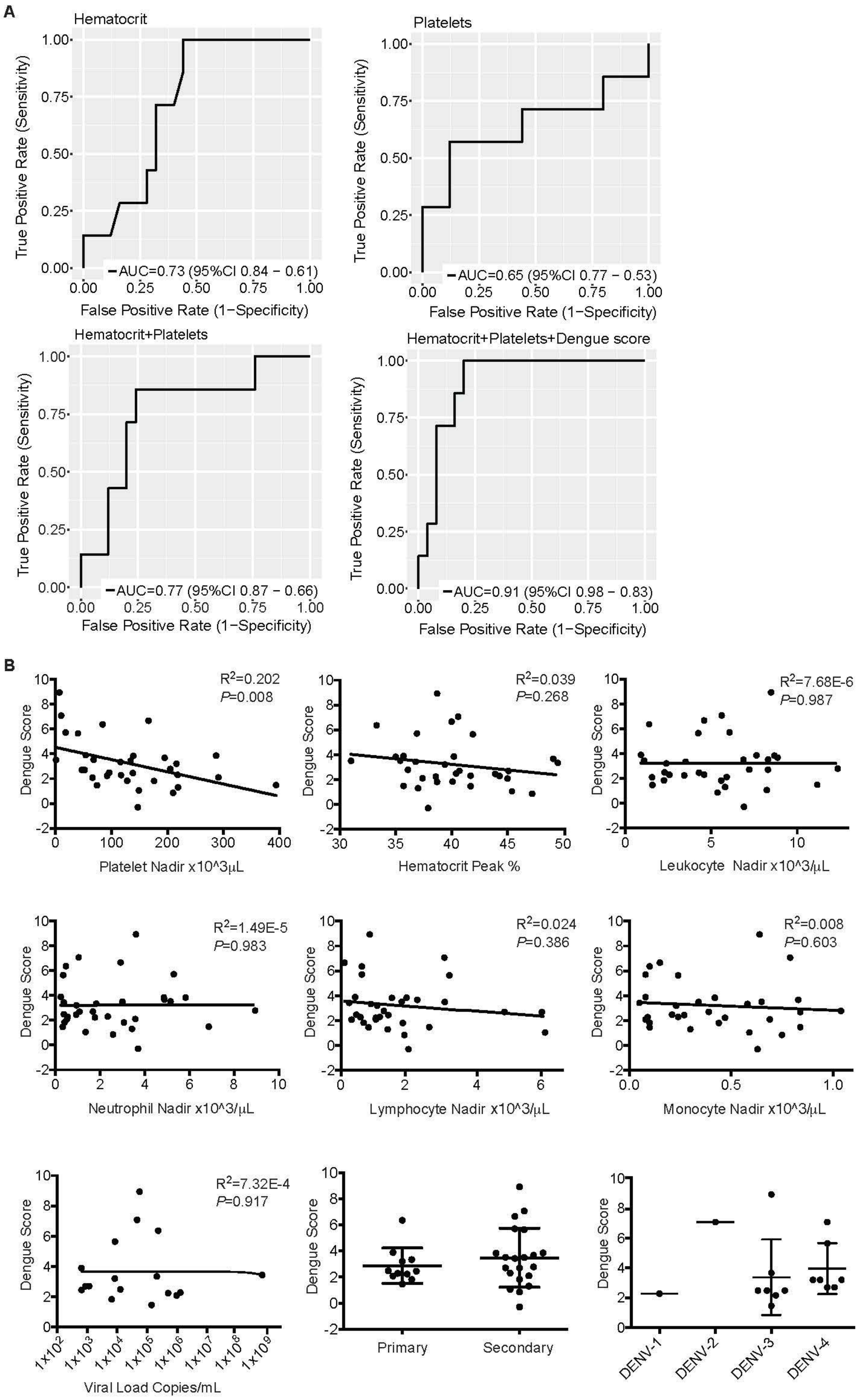
A
Hematocrit
True Positive Rate (Sensitivity)
False Positive Rate (1−Specificity)
AUC=0.73 (95%CI 0.84 − 0.61)
Platelets
AUC=0.65 (95%CI 0.77 − 0.53)
Hematocrit+Platelets
AUC=0.77 (95%CI 0.87 − 0.66)
Hematocrit+Platelets+Dengue score
AUC=0.91 (95%CI 0.98 − 0.83)
B
Dengue Score
R2=0.202
P=0.008
Platelet Nadir x10^3μL
R2=0.039
P=0.268
Hematocrit Peak %
R2=7.68E-6
P=0.987
Leukocyte Nadir x10^3/μL
R2=1.49E-5
P=0.983
Neutrophil Nadir x10^3/μL
R2=0.024
P=0.386
Lymphocyte Nadir x10^3/μL
R2=0.008
P=0.603
Monocyte Nadir x10^3/μL
R2=7.32E-4
P=0.917
Viral Load Copies/mL
Primary
Secondary
DENV-1
DENV-2
DENV-3
DENV-4

Figure S3. Routine laboratory parameters are ineffective in predicting development of severe dengue and at most part do not correlate with the dengue severity score, Related to Figure 2. A. ROC curves comparing patients with SD with patients with D and DWS based on hematocrit level (top left), platelet count (top right), hematocrit level and platelet count (bottom left) upon presentation in the Colombia cohort. Bottom right: ROC curve comparing patients with SD with patients with D and DWS based on the combination of the 20-gene set with hematocrit level and platelet count upon presentation in the Colombia cohort. B. Correlation of the dengue severity score with nadir platelet count, peak hematocrit, nadir of total leukocytes, neutrophils, lymphocytes, monocytes, viral load in serum, prior exposure to dengue, and dengue serotype via linear regression analysis. Mean±SD are shown in the lower mid and right panels.

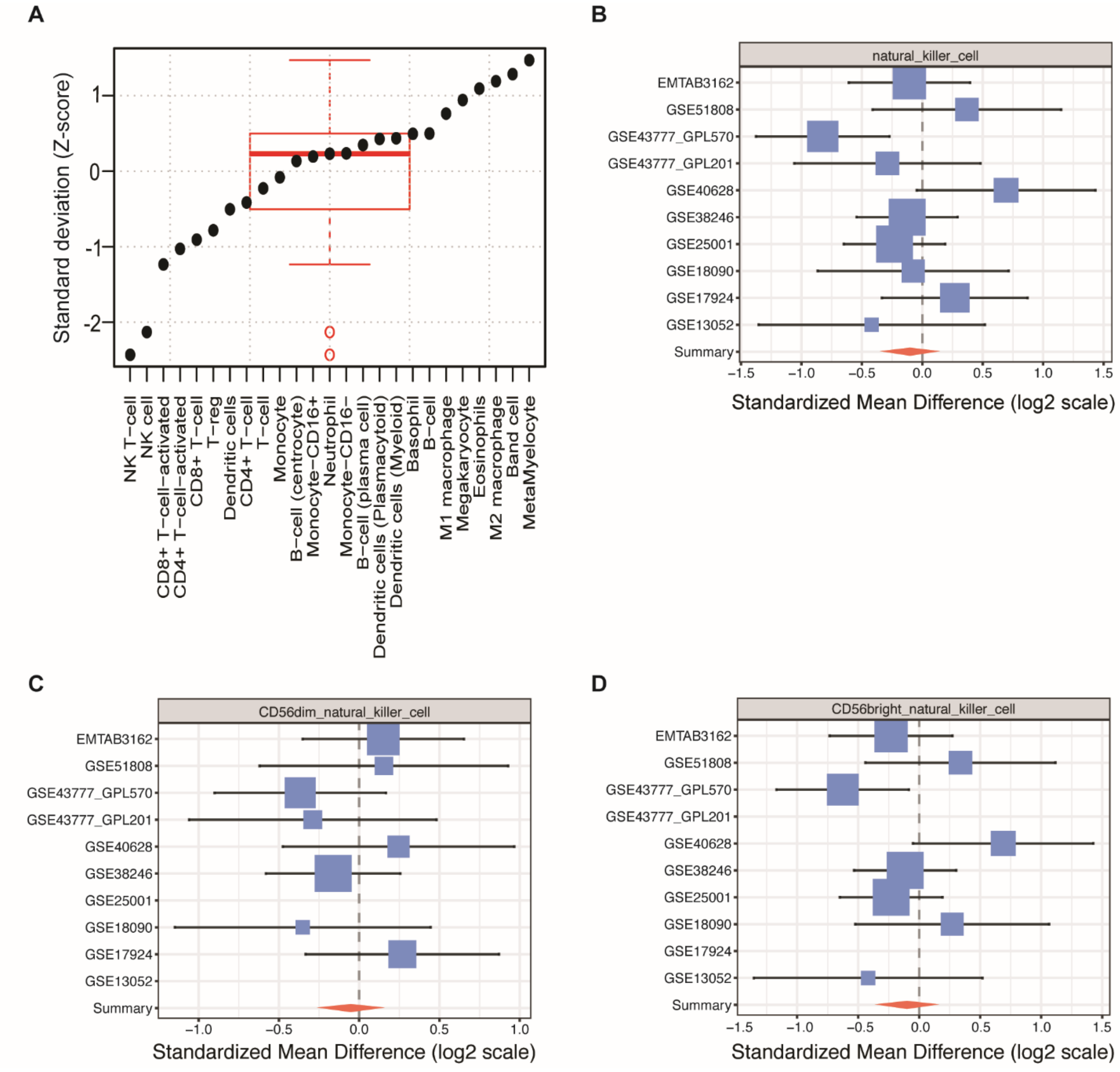

Figure S4. Enrichment profiles of the 20 differentially expressed genes in publicly available sorted-cell gene expression profiles and meta-analysis of cell-mixture deconvolution, Related to Figure 1. A. Y-axis shows standard deviations from the mean. Standardized enrichment scores (z scores, dots) for human immune cell types for the 20-gene set and a box plot of z score distributions are shown. B-D. Forest plots of total NK (B), CD56 dim NK (C), and CD56 bright NK (D) cell numbers determined by meta-analysis of cell-mixture deconvolution using immunoStates of blood transcriptome profiles of uncomplicated versus severe dengue patients in the discovery and validation cohorts. X-axis represents standardized mean difference between DHF/DSS and DF. The size of the blue rectangles is inversely proportional to the standard error of mean in the study. Whiskers represent the 95% CI. Red diamonds represent overall, combined mean difference for a given gene. Width of the diamonds represents the 95% CI of overall combined mean difference. $p>0.4$, FDR>80% for all panels.

	Platform	Year	Reference	Use	Country	Sample	Age	Controls Used	Cases Used	Total Used	Total Available	Timing of samples	Day 0
GSE13052	GPL2700	2009	Long, HT	Discovery	Vietnam	Whole blood	Children	9	9	18	30	Day 4 from symptoms, at time of admission, Day 30	fever
GSE25001	GPL6104	2010	Hoang, LT	Discovery	Vietnam	Whole blood	Children/ Adults	69	32	101	112	Day 2-10, follow up, days until defervesence	fever
GSE38246	GPL15615	2012	Popper, SJ	Discovery	Nicaragua	PBMCs	Children	46	42	88	113	Days 2-12	fever
GSE43777	GPL201	2013	Sun, P	Discovery	Venezuela	PBMCs	Children/ Adults	88	7	95	269	Day 0-5, post-defervesence, convalescence	admission; fever <120 hrs
GSE43777	GPL570	2013	Sun, P	Discovery	Venezuela	PBMCs	Children/ Adults	29	26	55	269	Day 0-5, post-defervesence, convalescence	admission; fever <120 hrs
GSE51808	GPL13158	2014	Kwissa, M	Discovery	Thailand	Whole blood	Children/ Adults	18	10	28	56	Day 2-9 (avg D5), follow up	fever
E-MTAB-3162	GPL570	2012	Van de Weg	Discovery	Indonesia	Whole blood	Adults	31	30	61	76	Day 0-6	admission, but within 48 hr of fever
	**Sum**									**446**			
GSE17924	GPL4133	2010	Devignot, S	Validation	Cambodia	Whole blood	Children	16	32	48	48	Day 3-7, shock onset, sampling	fever
GSE18090	GPL570	2009	Nascimento	Validation	Brazil	PBMCs	Adults	8	10	18	26	Day 1-8	fever
GSE40628	GPL16021	2007	Simmons	Validation	Vietnam	Whole blood	Adults	4	14	18	34	Day 4-12, late follow up	fever
	**Sum**									**84**			

Table S1. Publicly available datasets used for the discovery and validation of the 20-gene set, Related to Figures 1 and 2.

	Gene Symbol	Gene name	Expression in SD	Function
1	DEFA4	Defensin Alpha 4	Over	Innate immunity (neutrophils), antimicrobial, cytotoxic
2	GYG1	Glycogenin 1	Over	Innate immunity, glucose metabolism (glycosyltransferase)
3	TOR3A	Torsin Family 3 Member A	Over	Interferon response
4	PTPRM	Protein Tyrosine Phosphatase, Receptor Type M	Under	Innate immunity, cell adhesion, tyrosine phosphatase, lipid metabolism
5	SPON2	Spondin 2	Under	Innate immunity, cell adhesion, opsonization
6	GRAP2	GRB2-Related Adaptor Protein 2	Under	Leukocyte-specific protein tyrosine kinase signaling, viral clearance and pathogenesis
7	CACNA2D2	Calcium Voltage-Gated Channel Auxiliary Subunit Alpha2 delta 3	Under	Innate immunity, calcium channel
8	CACNA2D3	Calcium Voltage-Gated Channel Auxiliary Subunit Alpha2 delta 3	Under	Calcium channel
9	TMEM63C	Transmembrane Protein 63C	Under	Calcium activated cation channel
10	AK5	Adenylate Kinase 5	Under	Nucleoside kinase
11	CHD3	Chromodomain Helicase DNA Binding Protein 3	Under	Chromatin remodeling, transcription regulation, interacts with DENV proteins
12	CX3CR1	C-X3-C Motif Chemokine Receptor 1	Under	Innate immunity, chemokine receptor, leukocytes adhesion and migration
13	TRERF1	Transcriptional Regulating Factor 1	Under	Transcription regulation
14	GBP2	Guanylate Binding Protein 2	Under	Innate immunity, interferon gamma signaling, GTPase, antiviral, oxidative killing
15	SERINC5	Serine Incorporator 5	Under	Amino acid synthesis, restricts HIV infectivity
16	SOX13	SRY-Box 13	Under	Transcription regulation, cell fate, ERK and WNT signaling
17	NCR3	Natural Cytotoxicity Triggering Receptor 3	Under	Innate immunity, NK cell receptor
18	ABI3	ABI Family Member 3	Under	Regulation of actin polymerization
19	C3orf18	Chromosome 3 Open Reading Frame 1	Under	Uncharacterized
20	ENPP5	Ectonucleotide Pyrophosphatase/Phosphodiesterase 5	Under	Transmembrane glycoprotein

Table S2. Over-expressed and under-expressed genes identified in the discovery cohort via the multi-cohort analysis, Related to Figure 1.

		Dengue (N=9)	Dengue with WS (N=17)	Severe dengue (N=8)
Age	Adult	8	9	6
	Child (<17 years)	1	8	2
Gender	Male	4	7	0
	Female	5	10	8
First sample day	Mean (range)	3.5 (0-5)	3.2 (0-8)	2.7 (1-4)
Dengue diagnostics	Positive NS1 Ag	4	10	5
	Positive DENV IgM	8	15	6
Dengue exposure	Primary	4	6	2
	Secondary	4	11	6
	Undetermined	1		
Dengue serotype	DENV-1	1		
	DENV-3	1	7	4
	DENV-4	1	2	1
	DENV-2+4		1	
	Unknown	6	7	3
Clinical manifestations	Shock N (%)	0 (0)	0 (0)	6 (17.6)
	Plasma leakage N (%)	0 (0)	3 (8.8)	4 (11.7)
	Severe organ damage N (%)	0 (0)	0 (0)	6 (17.6)
	Bleeding N (%)	0 (0)	8 (23.5)	4 (11.7)
	Thrombocytopenia N (%)	1 (2.9)	8 (23.5)	6 (17.6)
	Hemoconcentration N (%)	3 (8.8)	8 (23.5)	3 (8.8)

Table S3. Colombia cohort. Demographic, clinical, and laboratory characteristics of study population, Related to Figure 2.

Patient num.	Shock criteria	Pulse peak	Pulse Pressure nadir	MAP nadir	Vasoactive support	Transfusion	Ventilatory support	Severe organ damage	Hemorrhagic manifestations	Fluid accumulation	Thrombocytopenia	Dengue severity score
	0=None<br>1= Weak Pulse<br>2= Cold/Clammy skin<br>3= Restlessness<br>4= Hypotension (<65mmHg)		mmHg	mmHg							<100,000	
1-001	1,2,3,4	120	38	56	+	+	+	+	-	+	+	5.72
1-002	1,2,4	140	29	50.6	+	+	+	+	+	+	+	8.94
1-010	1.4	104	25	44.3	-	-	-	+	+	+	+	3.90
1-013	4	98	19	62.6	-	-	-	+	+	-	+	5.65
1-017	0	100	23	71.6	-	-	-	-	+	-	+	3.50
1-026	4	85	35	63	-	-	-	+	+	+	-	3.44
1-029	2.4	93	29	44.6	-	-	-	-	-	-	-	6.67
1-036	0	99	22	78.6	-	-	-	+	-	-	+	(degraded RNA)

Table S4. Severe dengue patients in the Colombia cohort, Related to Figure 2**.**

Table S5. Colombia cohort. Demographic and clinical characteristics of study population, Related to Figure 2. Please, see separate Excel file.

	Dataset	Sensitivity	Specificity
Discovery	GSE13052gpl2700	1.00	0.778
	GSE25001gpl6104	0.938	0.290
	GSE38246gpl15615	0.952	0.326
	GSE43777gpl201	1.00	0.670
	GSE43777gpl570	0.962	0.690
	GSE51808gpl13158	0.900	0.556
	EMTAB3162gpl570	0.933	0.323
	Mean	0.955	0.519
	SD	0.036	0.203
Validation	GSE17924	0.969	0.250
	GSE18090	1.00	0.250
	GSE40628	0.929	0.500
	Mean	0.966	0.333
	SD	0.035	0.144
Colombia	D.DWS.SD	1.00	0.760
	DWS.SD	1.00	0.688
	DF.DHF.DSS	1.00	0.792

Table S6. Sensitivity and specificity of the 20-gene set in the pre-existing cohorts and new Colombia cohort, Related to Figures 1 and 2.
